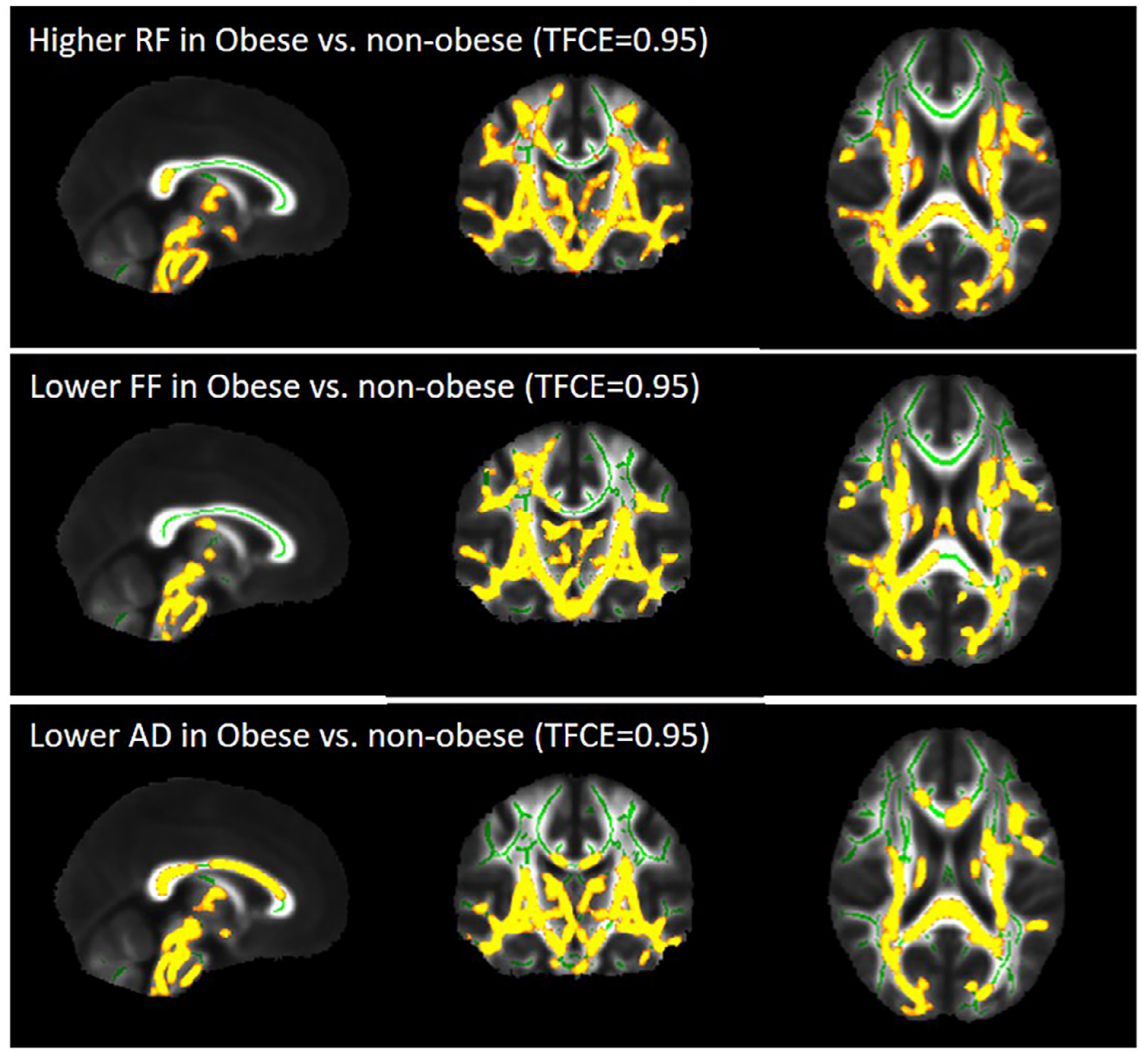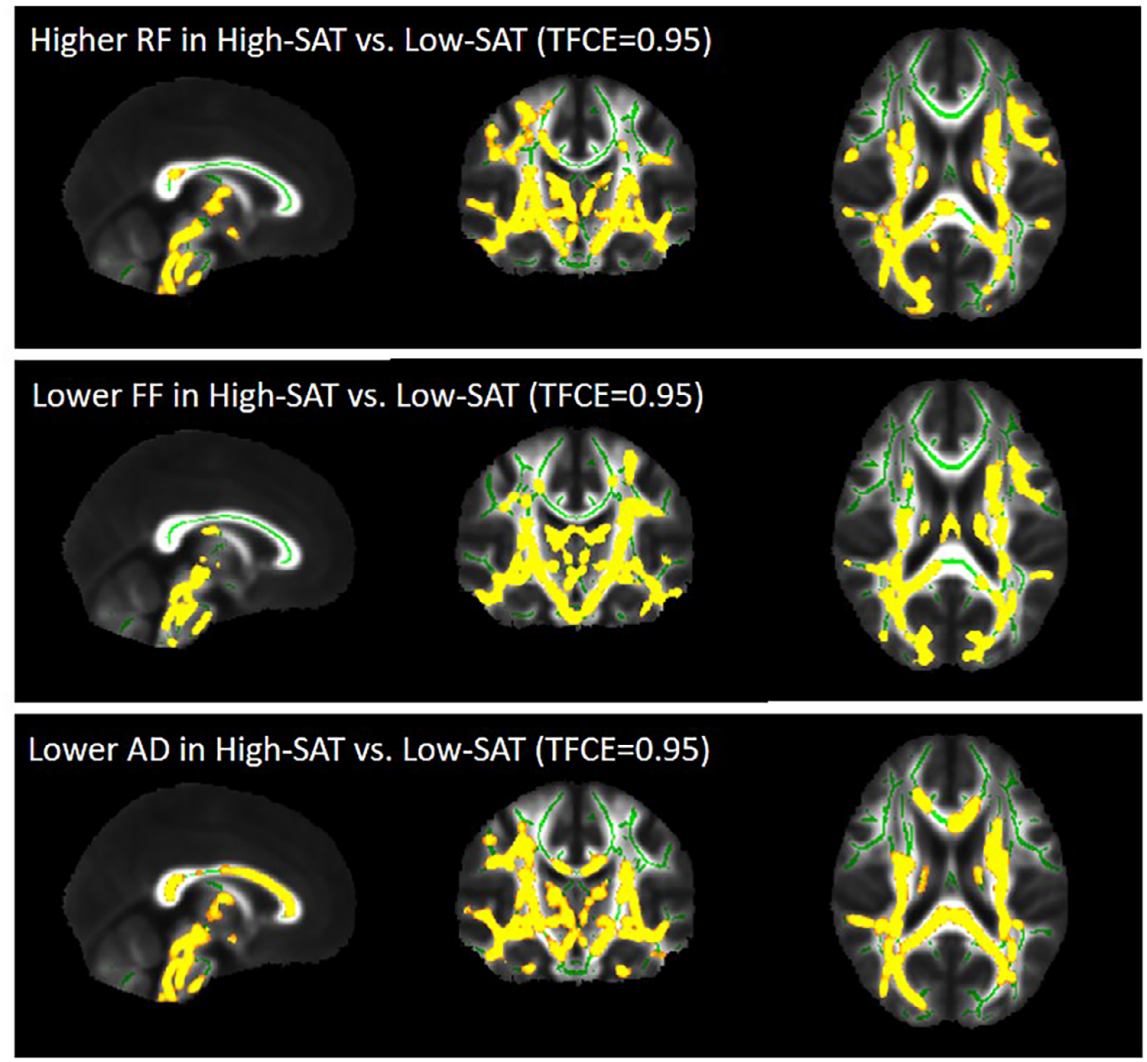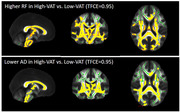# Diffusion Basis Spectrum Imaging in Midlife Obesity: Associations with Abdominal Adipose Tissue

**DOI:** 10.1002/alz.089979

**Published:** 2025-01-09

**Authors:** Mahsa Dolatshahi, Paul K. Commean, Farzaneh Rahmani, Caitlyn Nguyen, LaKisha Lloyd, Sara Hosseinzadeh Kasani, Bettina Mittendorfer, Claude Sirlin, Sheng‐Kwei Song, Tammie L.S. Benzinger, Joseph E. Ippolito, John C. Morris, Cyrus A. Raji

**Affiliations:** ^1^ Mallinckrodt Institute of Radiology, Washington University in St. Louis, St. Louis, MO USA; ^2^ Washington University in Saint Louis, Saint Louis, MO USA; ^3^ Missouri University School of Medicine, Columbia, MO USA; ^4^ University of California, San Diego, La Jolla, CA USA; ^5^ Washington University in St. Louis, St. Louis, MO USA; ^6^ Knight Alzheimer Disease Research Center, St. Louis, MO USA; ^7^ Washington University in St. Louis, School of Medicine, St. Louis, MO USA; ^8^ Washington University in St. Louis School of Medicine, St. Louis, MO USA

## Abstract

**Background:**

Obesity and abdominal adiposity in midlife are shown to increase the risk of Alzheimer disease. However, it is not clear whether midlife adiposity is associated with increased neuroinflammation. We aimed to investigate the associations of obesity, BMI of 30 kg/m^2^ or higher, and abdominal visceral and subcutaneous adipose tissue (VAT and SAT) with brain histology, using diffusion basis spectrum imaging (DBSI) analysis;

**Method:**

In total, 54 cognitively normal middle‐aged subjects (50.46±6.19 years, male: 21 (38.9%), obesity: 32 (59.3 %), BMI: 32.18±6.99 kg/m^2^) underwent brain and abdominal 3T MRI. Abdominal VAT and SAT were semi‐automatically segmented using VOXel Analysis Suite (Voxa). A DBSI scheme with a total of 98 diffusion samplings was acquired followed by movement and eddy current correction and brain tissue extraction in FSL. DBSI maps including fractional anisotropy (FA, overall integrity), axial diffusivity (AD, axonal injury), radial diffusivity (RD, myelin loss), restricted fraction (RF, inflammation cellularity), hindered fraction (HF, extracellular edema), and fiber fraction (FF, axonal density) were generated using in‐house software scripted in MATLAB. DBSI‐derived maps were processed using a tract‐based spatial statistics (TBSS) pipeline for whole‐brain white matter voxel‐wise analyses. Using the Randomize tool from FSL, the difference between obese vs. non‐obese, high‐VAT vs. low‐VAT, and high‐SAT vs. low‐SAT groups were investigated for each DBSI‐derived skeleton, with age and sex as covariates, and a threshold of 0.05 for false‐discovery rate. The sex differences were further investigated;

**Result:**

Lower FF and AD and higher RF in widespread white matter areas were observed in the obese vs. non‐obese group, and in the high‐SAT vs. low‐SAT group. Lower AD and higher RF were observed for the high‐VAT vs. low‐VAT group. All of these differences were only significant in females, not males, but higher RF in obese vs. non‐obese was observed both in males and females;

**Conclusion:**

Our data support lower axonal density and fiber integrity, as well as higher inflammation cellularity in cognitively normal, middle‐aged obese individuals, and those with high VAT and high SAT, especially in females. Overall, our data suggest the differential role of visceral and subcutaneous abdominal fat in promoting neuroinflammation and axonal damage.